# Characterization of dysbiosis of the conjunctival microbiome and nasal microbiome associated with allergic rhinoconjunctivitis and allergic rhinitis

**DOI:** 10.3389/fimmu.2023.1079154

**Published:** 2023-03-20

**Authors:** Yuan Wang, Xuan Li, Shuntong Gu, Junhong Fu

**Affiliations:** ^1^ Clinical College of Ophthalmology, Tianjin Medical University, Tianjin, China; ^2^ Tianjin Eye Hospital, Tianjin Key Laboratory of Ophthalmology and Visual Science, Tianjin Eye Institute, Tianjin, China; ^3^ Department of Otolaryngology-Head & Neck Surgery, Tianjin TEDA Hospital, Tianjin, China; ^4^ Nankai University Affiliated Eye Hospital, Nankai University, Tianjin, China; ^5^ Department of General Surgery, Tianjin Fifth Central Hospital, Tianjin, China; ^6^ Department of Vascular Surgery, Tianjin Medical University General Hospital, Tianjin, China; ^7^ Department of Ophthalmology, Tianjin TEDA Hospital, Tianjin, China

**Keywords:** allergic rhinoconjunctivitis, allergic rhinitis, microbiome, conjunctival, nasal, 16s rRNA amplicon sequencing

## Abstract

**Background:**

Allergic rhinoconjunctivitis (ARC) and allergic rhinitis (AR) are prevalent allergic diseases. People are becoming increasingly aware of the impact of microbial disorders on host immunity and allergic diseases. Studies have demonstrated an association between allergic diseases and the microbiome, but much remains unknown. We assessed changes in the conjunctival microbiome and nasal microbiome in patients with ARC or AR.

**Methods:**

Conjunctival swabs and nasal swabs were collected from each participant for 16S rRNA amplicon sequencing. Bacterial communities were analyzed.

**Results:**

Forty patients with ARC, 20 patients suffering from AR, and 34 healthy controls (HCs) were recruited. This study found the abundance of conjunctival microbiome in patients with ARC or AR was significantly lower than that in HCs. The diversity of conjunctival microbiome in patients with AR was significantly lower than those in the other two groups. There is no significant difference in abundance of nasal microbiome between the three groups. The diversities of nasal microbiome in patients with ARC or AR were significantly lower than that in HCs. We found significant differences in microbiota compositions in patients with ARC or AR compared with those in HCs. However, no significant difference in microbiota compositions was found between patients with ARC and patients with AR. Microbiome functions in the ARC group and AR group were also altered compared with HCs.

**Conclusions:**

We revealed changes in the composition and function of the conjunctival microbiome and nasal microbiome of patients with ARC or AR, which suggests that there is a relationship between allergic conditions and the local microbiome.

## Introduction

Allergic rhinitis (AR) is an immune-mediated disease of the nose caused by hypersensitivity reactions, such as itching, sneezing, increased secretion and obstruction. Allergic conjunctivitis (AC) is an inflammatory disease of the conjunctiva caused mainly by an immunoglobulin (Ig)E-mediated mechanism (1). When allergic conjunctivitis and allergic rhinitis coexist, it can be diagnosed as allergic rhinoconjunctivitis (ARC) (1, 2). They are common allergic diseases and the incidence is increasing year by year. The worldwide prevalence of AR, AC, and ARC has been reported to be 28.3%, 15.9%, and 12.3%, respectively (3).

ARC and AR are caused primarily by sensitization to specific aeroallergens, and result in local synthesis of IgE and histamine release (4, 5). As a result, patients experience inflammation of the upper respiratory mucosa and conjunctiva, which leads to repeated or chronic sneezing, rhinorrhea, nasal congestion, red eyes, as well as itching of the nose and eyes (6). Medications provide temporary relief, but ARC and AR are incurable and have a serious impact on quality of life. Therefore, understanding the pathophysiology of AR and ARC is needed to develop better management strategies.

The lesion sites of ARC are ocular surface and nasal cavity. The ocular surface consists of the conjunctiva, cornea, sclera and tear film. They are continuous with the skin of the eyelid and exposed to the environment, and form the ocular surface microenvironment of the eye. A normal surface microenvironment of the eye is essential for the health of the ocular surface. Microorganisms on the ocular surface may originate from the body or external environment. Most people have a relatively stable microbial community on the ocular surface (7), including fungi, bacteria and viruses, with bacteria being the most abundant and important (8, 9).

The nasal cavity is a physical transition from a space of constant contact with the outside world to a highly regulated and protected interior space, where innate microbes play a key part in health and disease. Respiratory microbiome may be the gatekeeper against respiratory pathogen colonization. It is also probable that the respiratory microbiota may participate in the maturation and maintenance of respiratory physiological and immune homeostasis (10). It was found that nasal microbes play a key part in the pathogenetic mechanism of AR. As early as 1989, there was a hygiene hypothesis that exposure to nasal pathogens could cause increased tolerance and a significant reduction in allergic rhinitis in children (11).

Previously, there have been controversies about the impact on ocular health due to the low biomass of the conjunctival microbiota (7, 8, 12). Unlike the rest of the body (mouth, gut, skin), the eye surface is considered to be quite sparse, with only the occasional microorganism entering due to the powerful antibacterial properties of the tear membrane and the continuous mechanical action of eyelids (13). Microbiology research based on traditional cultivation—a technique of artificially growing and multiplying bacteria—can be used to isolate low-diversity microbes from the eye surface, but this method is susceptible to various physicochemical factors and has poor stability. Not using cultivation methods allows analyses of the microbiome based on differences in 16S ribosomal RNA gene sequences. Moreover, these methods have been used to characterize the normal ocular microbiota (14–19). Thuy Doan found the core constituents of the conjunctival microbiome appear relatively consistent between individuals, and are dominated by the four genera of *coagulase-negative Staphylococci, Diphtheroids, and Propionibacteria, and Streptococci* (14). Qunfeng Dong found 12 genera—*Pseudomonas, Propionibacterium, Bradyrhizobium, Corynebacterium, Acinetobacter, Brevundimonas, Staphylococci, Aquabacterium, Sphingomonas, Streptococcus, Streptophyta, and Methylobacterium*—were ubiquitous among the analyzed cohort and represented the putative “core” of conjunctival microbiota (18). The role of the conjunctival microbiome in healthy ocular surfaces and different types of ocular diseases is of increasing concern. Interference with the microbiome, such as allergies, illnesses or exposure to drugs, can also pose risks to ocular health (20). The microbial community of the ocular surface seems more diverse than that reported previously, with alterations in the ocular microbiome being found in several ocular disorders (9, 16).

The pathogenesis of allergic diseases is complex and influenced by a combination of environmental and genetic factors. The interaction between the adverse biological behavior of the microbiome and allergic reactions to allergen exposure plays an important part (21). Evidence suggests that dysfunctional local microbial communities (gut, respiratory tract, and skin) could be related to allergy risk (22, 23). Recent studies have shown that gut microbes play an important part in the pathogenesis of several allergic diseases, including asthma and eczema (24). Although microbial biomass is lower in other parts of the body than that in the gut, scholars have sought to study the microbial composition of other body parts to link more directly to extra-intestinal diseases (25, 26).

The relationship between ARC and AR and the microbiome of the eye and nose is unexplored. Investigating the relationship between allergic inflammation and the ocular surface and the microbiota in the upper respiratory tract is key to understanding the mechanisms underlying ARC and AR, and providing potential treatment strategies.

In the present study, the conjunctival microbiome and nasal microbiome from healthy controls (HCs) and patients suffering from ARC or AR were analyzed using 16S rRNA amplicon sequencing.

## Methods

### Ethical approval of the study protocol

The study was conducted in Tianjin TEDA Hospital (Tianjin, China). The study protocol was approved (2022-02) by the ethics committee of TEDA Hospital. All individuals provided written informed consent before participating in the study.

### Exclusion criteria

The exclusion criteria were: (i) active ocular inflammation or dry eye; (ii) having ARC or AR along with sinusitis, nasal polyps, or non-allergic rhinitis; (iii) suffering from autoimmune disease or cancer; (iv) use of eye drops, nasal sprays, topical/systemic corticosteroids, antibiotics, or immunomodulatory medications within the previous 3 months; (v) currently pregnant or lactating; (vi) not possible (for any reason) to cooperate with the research protocol.

### Participants and study design

Ninety-four participants (34 HCs and 60 patients) aged 18–60 years were recruited from Tianjin TEDA Hospital between January and March 2022. Patients whose previous test results suggested they were allergic to dust mites and/or mold were recruited.

People suspected of having ARC or AR were diagnosed using the Allergic Rhinitis and its Impact on Asthma guideline (27) and Documento dE Consenso sobre Conjuntivitis Alérgica (28). The Rhinoconjunctivitis Quality of Life Questionnaire and Rhinoconjunctivitis Daily Symptom Score were used for all patients. Patients were examined by the same otolaryngologist and the same ophthalmologist. Nasal and conjunctival conditions were recorded.

All patients had more than two symptoms of sneezing, nasal itching, watery nasal discharge, and nasal congestion. Physical examination showed a pale nasal mucosa, edema, and watery secretions from the nose. Some patients also had ocular symptoms such as tears, itching, and redness. Mild-to-moderate hyperemia and edema were observed on the conjunctiva by examination using a slit lamp. These patients were classified as the ARC group. Those with nasal symptoms but no ocular symptoms were classified as the AR group. These 60 patients were divided into two groups: 40 with ARC and 20 with AR.

### Sample collection

Swabs were taken from the conjunctiva and nose. We sampled: 80 eyes and 80 nasal cavities of 40 patients with ARC; 40 eyes and 40 nasal cavities of 20 patients with AR; 68 eyes and 68 nasal cavities of 34 HCs.

Using a sterile cotton swab, we wiped the conjunctival sac from the medial side to the lateral side of the inferior fornix (being very careful to not to touch the eyelids). This procedure was repeated thrice. Nasal samples were collected from the surface of inferior turbinate with a sterile swab under guidance by a nasal endoscope. Then, the swabs were placed in sterile tubes. Samples were stored temporarily at 0°C and then transferred to a deep freezer (−80°C) until they were sent to Novogene (Beijing, China).

### DNA extraction, polymerase chain reaction amplification, and 16S rRNA gene amplicon sequencing

Genome-wide DNA from samples was extracted using hexadecyl trimethylammonium bromide and cetyltrimethylammonium bromide (CTAB). The concentration and purity of DNA were monitored on 1% agarose gels. According to the concentration, DNA was diluted to 1 ng/μL with sterile water. If swabs from both sides had been collected successfully, only samples with high DNA yield were collected. The V3–V4 hypervariable region of the 16S rRNA gene was amplified using the fusion primers 341F (5’-ACTCCTACGGGAGGCAGCAG-3’) and 806R (5’-GGACTACHVGGGTWTCTAAT-3’) (Sangon Biotech, Shanghai, China). All PCR mixtures contained Phusion^®^ High-fidelity PCR Master Mix (15 μL; New England Biolabs, Ipswich, MA, USA), each primer (0.2 μM), and target DNA (10 ng). Cycling conditions comprised an initial denaturation step at 98°C for 1 min, followed by 30 cycles at 98°C (10 s), 50°C (30 s) and 72°C (30 s), and a final extension (5 min) at 72°C. We mixed an equal volume of 1× loading buffer containing SYB green with PCR products and undertook electrophoresis on 2% agarose gel for DNA detection. PCR products were mixed in equal proportions, and the Universal DNA PCR Purification Kit (catalog number: DP214; TianGen, Beijing, China) was used to purify the mixed PCR product. Following manufacturer recommendations, sequencing libraries were generated with Next Ultra DNA Library Prep Kit for Illumina (E7370L; New England Biolabs). The quality of libraries was assessed on a 5400 system (Agilent Technologies, Santa Clara, CA, USA) and quantified by real-time PCR (1.5 nM). Finally, libraries were sequenced on a NovaSeq™ platform (Illumina, San Diego, CA, USA) and 250-bp paired-end reads. Raw data have been deposited in the National Center for Biotechnology Information Sequence Read Archive database (BioProject number: PRJNA899122).

### Data analyses

FLASH 1.2.11 (29) is a rapid and accurate analytical tool. It is designed to merge paired-end reads if at least some of the reads overlap with the reads generated from the opposite end of the same DNA fragment. The splicing sequences are called “raw tags”. Quality filtering of raw tags was carried out using fastp 0.20.0 to obtain high-quality clean tags. The latter were compared with a reference database (Silva database for 16S) using Vsearch 2.15.0 to detect chimera sequences. The latter were removed to obtain effective tags (30).

For the effective tags we obtained, denoising was undertaken with the “DADA2” module or “deblur” module in QIIME2-202006 (31) to obtain initial amplicon sequence variants (ASVs) (default: DADA2). Then, ASVs with abundance <5 were filtered out (32). Species annotation was undertaken using QIIME2. Multiple sequence alignment was done using QIIME2 to study the phylogenetic relationship of each ASV and differences in dominant species among different samples (groups). The absolute abundance of ASVs was normalized using a standard of the sequence number corresponding to the sample with the least number of sequences. Subsequent analysis of alpha diversity and beta diversity was undertaken based on output normalized data.

We wished to analyze the uniformity, richness, and diversity of bacterial communities in a sample. Alpha diversity was calculated from seven indices in QIIME2: “Observed-OTUs”, “Chao1”, “Simpson”, “Shannon”, “Good’s coverage”, and “Pielou-e”. Two indicators were selected to determine community richness: Chao1 (Chao1 Index) and Observed-OTUs (number of observed species). Two indices were used to determine community diversity: Simpson (Simpson Index) and Shannon (Shannon Index). Good’s coverage was applied to calculate the depth of sequences. Pielou’s Evenness Index was used to calculate the evenness of a species.

Beta diversity was calculated based on weighted and unweighted unifrac distances in QIIME2. Beta diversity was employed to evaluate the complexity of community composition and compare differences between samples.

Cluster analysis was undertaken by applying principal component analysis. We used the “ade4” and “ggplot2” packages in R 3.5.3 (R Institute for Statistical Computing, Vienna, Austria) to reduce the dimensionality of raw variables.

Principal coordinate analysis (PCoA) was used to obtain principal coordinates and visualize sample differences in complex multidimensional data. A previously obtained matrix of weighted or unweighted unit frame distances between samples was converted into a new set of orthogonal axes in which the maximum coefficient of variation was expressed by the first principal coordinate, the second maximum coefficient by the second principal coordinate, and so on.

The “adonis” and “anosim” functions in QIIME2 were employed to investigate the significance of differences in population structure between groups. The Student’s *t*-test was done using R 3.5.3 to identify significantly different species at each taxonomic level. Linear discriminant analysis effect size (LEfSe) (threshold of LDA score = 4) was carried out using LEfSe 1.0 to identify biomarkers.

### Functional analyses

We wished to study the community function in samples and identify different community functions in different groups. Functional annotation was done using PICRUSt2 2.1.2-b. Functional differences between three groups were examined by one-way ANOVA.

## Results

### Clinical characteristics, sample groupings, sequencing statistics, and data preprocessing

The study cohort comprised 40 individuals with ARC, 20 individuals with AR, and 34 HCs. There were no significant differences in age, sex, or disease course among the three groups. The characteristics and detailed demographic information of participants at baseline are presented in **Table 1**.

**Table 1:** Characteristics of the three groups.

characteristic	HC	ARC	AR	P-value
Subjects(no.)	34	40	20	
Age(y)	38.47 ± 11.27	40.22 ± 9.84	38.40 ± 8.86	0.751
Sex ratio(M/F)	13/21	15/25	6/14	0.809
Course(y)	–	8.17 ± 7.292	8.55 ± 7.258	0.687

HC, healthy controls; ARC, allergic rhinoconjunctivitis; AR, allergic rhinitis.

**Figure 1:**
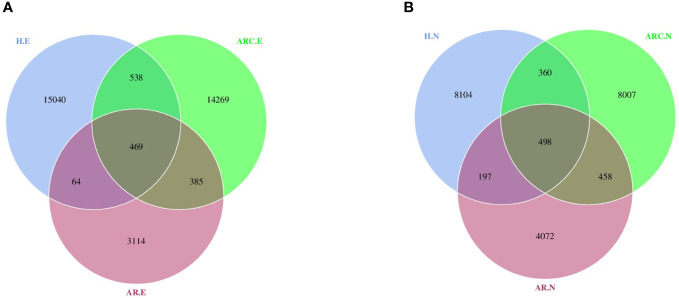
Venn diagram showing overlaps of the operational taxonomic units (OTUs) among the three groups. **(A)** Venn diagram of conjunctival microbiome. **(B)** Venn diagram of nasal microbiome. HC.E, eye of healthy controls; HC.N, nose of healthy controls; ARC.E, eye of allergic rhinoconjunctivitis; ARC.N, nose of allergic rhinoconjunctivitis; AR.E, eye of allergic rhinitis; AR.N, nose of allergic rhinitis.

With respect to the conjunctival microbiome, a Venn diagram showed that 469 ASVs were shared among the three groups, whereas 14269 were unique for ARC, 3114 were unique for AR, and 15040 were specific for HCs. With regard to the nasal microbiome, a Venn diagram showed that 498 ASVs were shared among the three groups, whereas 8007 were unique for ARC, 4072 were unique for AR, and 8104 were specific for HCs (**Figures 1A, B**).

**Figure 2:**
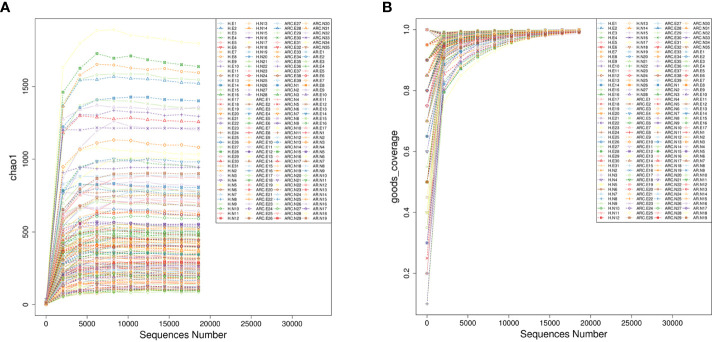
Alpha rarefaction represented by Chao1 index **(A)** and goods-coverage index **(B)**. The horizontal axis represents the amount of sequencing data, and the vertical axis represents the corresponding alpha diversity index.

Rarefaction curves indicated that the microbial abundance of samples was close to saturation if applied to sequencing depths (**Figures 2A, B**), which was sufficient to determine most bacterial-community members in each microbiome.

### Alpha diversity in the conjunctival microbiome and nasal microbiome of samples

The Chao1 Index, Observed-OTUs, Shannon Index, and Simpson Index were calculated to measure differences in taxonomic diversity among groups. This study found the abundance of conjunctival microbiome in patients with ARC or AR was significantly lower than that in HCs. The diversity of conjunctival microbiome in patients with AR was significantly lower than those in the other two groups. There is no significant difference in abundance of nasal microbiome between the three groups. The diversities of nasal microbiome in patients with ARC or AR were significantly lower than that in HCs.

**Figure 3:**
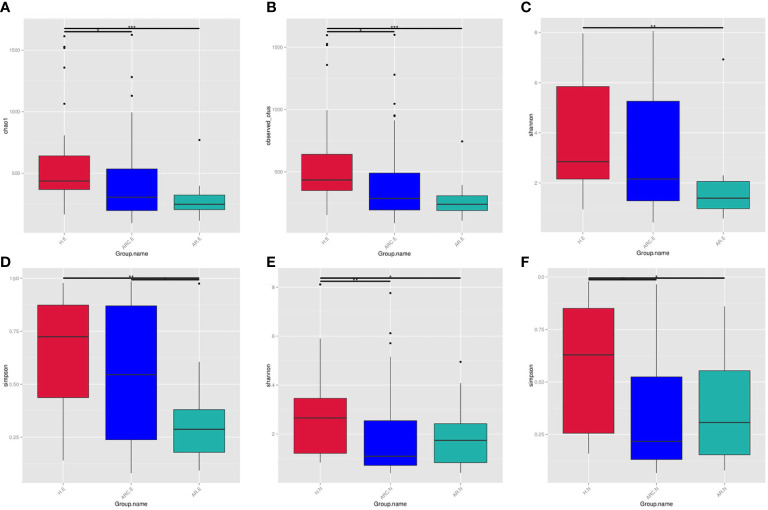
The alpha diversity of conjunctival microbiome represented by Chao1 index **(A)**, Observed-otus index **(B)**, Shannon index **(C)**, and Simpson index **(D)**. The alpha diversity of nasal microbiome represented by Shannon index **(E)** and Simpson index **(F)**, *p < 0.05, **p < 0.01, ***p < 0.001.

With respect to the conjunctival microbiome, the Chao1 Index and Observed-OTUs tended to be lower with AR (Chao1 Index: *p* < 0.001; Observed-OTUs: *p* < 0.001) and with ARC (Chao1 Index: *p* = 0.015; Observed-OTUs: *p* = 0.016) compared with HCs (**Figures 3A, B**). A lower Shannon Index was found in patients with AR (*p* = 0.002) compared with HCs (**Figure 3C**). A lower Simpson Index was found in patients with AR (*p* = 0.029 *vs*. eye of allergic rhinoconjunctivitis (ARC.E) and *p* = 0.002 *vs*. eye of healthy controls (HC.E)) (**Figure 3D**) compared with the other two groups.

With regard to the nasal microbiome, there was no significant difference in the Chao1 Index or Observed-OTUs among the three groups (Chao1 Index: *p* = 0.758 nose of allergic rhinoconjunctivitis (ARC.N) *vs*. nose of healthy controls (HC.N), *p* = 0.918 nose of allergic rhinitis (AR.N) *vs*. HC.N, *p* = 0.691 ARC.N *vs*. AR.N; Observed-OTUs: *p* = 0.276 ARC.N *vs*. HC.N, *p* = 0.592 AR.N *vs*. HC.N, *p* = 0.675 ARC.N *vs*. AR.N). The Shannon Index and Simpson Index tended to be lower with ARC and AR compared with HCs (Shannon Index: *p* = 0.001 ARC.N *vs*. HC.N, *p* = 0.016 AR.N *vs*. H.N, **Figure 3E**; Simpson Index: *p* < 0.001 ARC.N *vs*. HC.N and *p* = 0.006 AR.N *vs*. HC.N, **Figure 3F**). However, no significant difference in the Shannon Index or Simpson Index was found between the ARC group and AR group (Shannon Index: *p* = 0.663; Simpson Index: *p* = 0.771).

When comparing the conjunctival microbiome and nasal microbiome of the same group, the Observed-OTUs of the conjunctival microbiome was significantly higher than that of the nasal microbiome in HCs (*p* = 0.013) and ARC group (*p* = 0.0293). The Simpson Index of the conjunctival microbiome was significantly higher than that of nasal microbiome of the ARC group (*p* = 0.001).

### Beta diversity of the conjunctival microbiome and nasal microbiome in samples

This study found significant differences in nasal and conjunctival microbiota compositions in patients with ARC or AR compared with those in HCs.

**Figure 4:**
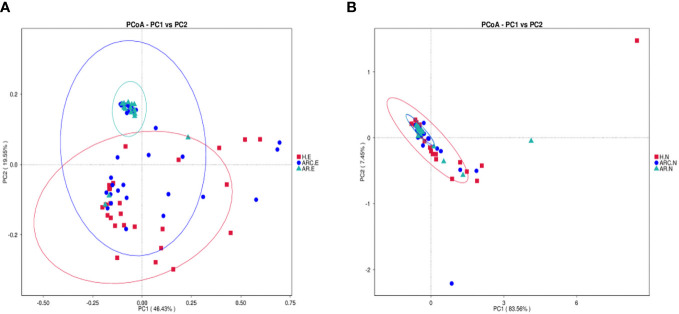
Principal Coordinate Analysis (PCoA) of beta diversity based on the weighted UniFrac distances. **(A)** PCoA of conjunctival microbiome among ARC group, AR group, and healthy controls. **(B)** PCoA of nasal microbiome among ARC group, AR group, and healthy controls.

PCoA plots reflect the beta diversity of the microbiota. We wanted to obtain master coordinates and show differences in samples using complex multidimensional data. Obvious clustering of conjunctival microbiome was not observed in the PCoA plots between HCs and the other two groups. Clustering of nasal microbiome was observed between the ARC group and AR group (**Figures 4A, B**).

The weighted-unifrac distance in the “anosim” package was used to ascertain significant differences in community structure between groups. There were significant differences between the ARC group and HCs (*p* = 0.005) and between the AR group and HCs (conjunctiva: *p* = 0.005; nose: *p* = 0.04). However, there was no significant difference between the ARC group and AR group (conjunctiva: *p* = 0.801; nose: *p* = 0.448).

### Characterization of the conjunctival microbiome and nasal microbiome in the three groups

**Figure 5:**
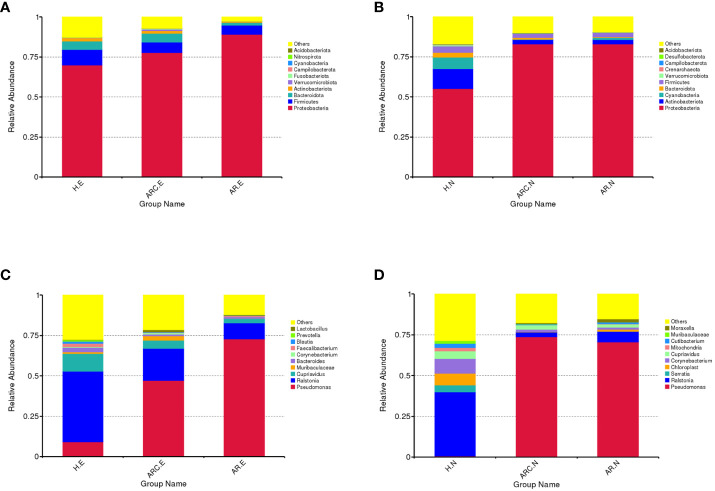
Box plots of the phylum and genus taxonomic levels in ARC, AR and healthy controls. **(A)** Top 10 phyla of conjunctival microbiome in the three groups. **(B)** Top 10 phyla of nasal microbiome in the three groups. **(C)** Top 10 genera of conjunctival microbiome in the three groups. **(D)** Top 10 genera of nasal microbiome in the three groups.

In the three groups (**Figures 5A, B**), the top 10-phyla of the conjunctival microbiome were *Proteobacteria*, *Firmicutes*, *Bacteroidota*, *Actinobacteriota*, *Verrucomicrobiota*, *Fusobacteriota*, *Campilobacterota*, *Cyanobacteria*, *Nitrospirota*, and *Acidobacteriota*. The most abundant phylum was *Proteobacteria* (HCs, 69.80%; ARC, 77.68%; AR, 89.16%). Other phyla accounting for >1% of OTUs were *Firmicutes* (HCs, 9.72%; ARC, 6.52%; AR, 5.61%), *Bacteroidota* (HCs, 5.34%; ARC, 5.53%; AR, 1.77%), and *Actinobacteriota* (HCs, 2.06%; ARC, 1.70%). *Proteobacteria* was more abundant in the AR group compared with the ARC group (89.16% *vs*. 77.68%, *p* = 0.044) and HCs (89.16% *vs*. 69.80%, *p* = 0.005) (**Table 2**). The most abundant phylum of the nasal microbiome was *Proteobacteria* (HCs, 55.09%; ARC, 82.90%; AR, 82.98%). Other phyla accounting for >1% of OTUs were *Actinobacteriota* (HCs, 12.39%; ARC, 2.90%; AR, 2.79%), *Cyanobacteria* (HCs, 7.21%; ARC, 0.16%; AR, 1.25%), *Bacteroidota* (HCs, 3.10%; ARC, 1.00%; AR, 0.43%), and *Firmicutes* (HCs, 4.00%; ARC, 2.55%; AR, 2.77%) (**Table 3**). HCs had significantly lower abundance of *Proteobacteria* (*p* < 0.001) and significantly higher abundance of *Actinobacteriota* (*p* < 0.05) than patients with ARC or AR.

**Table 2:** The main components of conjunctival microbiome in the three groups at the Phylum level and Metastat analysis results.

Phylum	ARC.E Relative abundance(%)	AR.E Relative abundance(%)	HC.E Relative abundance(%)	P-value		
				ARC-HC	AR-HC	ARC-AR
Proteobacteria	77.68	89.16	69.80	/	0.005	0.044
Firmicutes	6.52	5.61	9.72	/	/	/
Bacteroidota	5.53	1.77	5.34	/	/	/
Actinobacteriota	1.70	0.71	2.06	/	0.026	/
Verrucomicrobiota	0.76	0.04	0.04	0.006	/	/
Fusobacteriota	0.45	0.01	0.05	/	/	0.008
Campilobacterota	0.16	0.03	0.01	0.012	/	/
Cyanobacteria	0.08	0.04	0.11	/	/	/
Nitrospirota	0.01	0.00	0.04	/	/	/
Acidobacteriota	0.05	0.01	0.05	/	/	/

HC, healthy controls; ARC, allergic rhinoconjunctivitis; AR, allergic rhinitis. / p>0.05.

**Table 3:** The main components of nasal microbiome in the three groups at the Phylum level and Metastat analysis results.

Phylum	ARC.N Relative abundance(%)	AR.N Relative abundance(%)	HC.N Relative abundance(%)	P-value		
				ARC-HC	AR-HC	ARC-AR
Proteobacteria	82.90	82.98	55.09	<0.001	<0.001	/
Actinobacteriota	2.90	2.79	12.39	0.030	0.033	/
Cyanobacteria	0.16	1.25	7.21	<0.001	/	/
Bacteroidota	1.00	0.43	3.10	/	/	/
Firmicutes	2.55	2.77	4.00	/	/	/
Verrucomicrobiota	0.09	0.04	0.62	/	/	/
Crenarchaeota	0.00	0.00	0.12	/	/	/
Campilobacterota	0.11	0.01	0.15	/	0.049	/
Desulfobacterota	0.11	0.03	0.05	/	/	/
Acidobacteriota	0.05	0.05	0.20	/	/	/

HC, healthy controls; ARC, allergic rhinoconjunctivitis; AR, allergic rhinitis. / p>0.05.

In the three groups (**Figures 5C, D**), the top-10 genera of the conjunctival microbiome were *Pseudomonas*, *Ralstonia*, *Cupriavidus*, *Muribaculaceae*, *Bacteroides*, *Corynebacterium*, *Faecalibacterium*, *Blautia*, *Prevotella*, and *Lactobacillus*. The top-three most prevalent candidates in patients with ARC or AR were the genera *Pseudomonas* (ARC, 47.00%; AR, 72.85%), *Ralstonia* (ARC, 19.90%; AR, 9.92%), and *Cupriavidus* (ARC, 5.13%; AR, 2.71%). However, the most abundant genus in HCs was *Ralstonia* (43.64%). Other genera accounting for >1% in HCs were *Cupriavidus* (11.16%), *Pseudomonas* (9.09%), *Bacteroides* (2.82%), *Faecalibacterium* (1.98%), and *Blautia* (1.24%). The abundance of *Pseudomonas* was markedly higher in the ARC group and AR group than that in HCs (47.00% *vs*. 9.09%, *p* < 0.001; 72.85% *vs*. 9.09%, *p* < 0.001). The abundance of *Ralstonia* was markedly higher in HCs compared with that in the ARC group and AR group (43.64% *vs*. 19.90%, *p* = 0.003; 43.64% *vs*. 9.92%, *p* = 0.002) (**Table 4**). The most abundant genera of the nasal microbiome in the ARC group and AR group were *Pseudomonas* (ARC, 73.64%; AR, 70.58%), *Ralstonia* (ARC, 3.02%; AR, 6.54%), *Cupriavidus* (ARC, 2.6%; AR,1.90%), *Corynebacterium* (ARC, 1.6%; AR, 1.26%), and *Moraxella* (ARC, 0.68%; AR, 1.79%). The most abundant genera of the nasal microbiome in HCs were *Ralstonia* (39.24%), *Corynebacterium* (9.14%), *Chloroplast* (7.2%), *Cupriavidus* (4.56%), *Serratia* (4.2%), *Cutibacterium* (2.48%), *Mitochondria* (2.07%), and *Muribaculaceae* (1.73%) (**Table 5**). The abundance of *Pseudomonas* was markedly higher in the ARC group and AR group than that in HCs (*p* < 0.001). The *abundance* of *Ralstonia* and *Serratia* was markedly higher in HCs compared with that in the other two groups.

**Table 4:** The main components of conjunctival microbiome in the three groups at the Genus level and Metastat analysis results.

Genus	ARC.E Relative abundance(%)	AR.E Relative abundance(%)	HC.E Relative abundance(%)	P-value		
				ARC-HC	AR-HC	ARC-AR
Pseudomonas	47.00	72.85	9.09	<0.001	<0.001	0.018
Ralstonia	19.90	9.92	43.64	0.003	0.002	0.020
Cupriavidus	5.13	2.71	11.16	/	/	/
Muribaculaceae	2.82	0.03	0.96	/	0.009	/
Bacteroides	0.92	0.99	2.82	/	/	/
Corynebacterium	0.78	0.07	0.40	/	0.022	/
Faecalibacterium	0.31	0.59	1.98	0.037	/	/
Blautia	0.36	0.26	1.24	/	/	/
Prevotella	0.10	0.10	0.96	0.025	/	/
Lactobacillus	1.32	0.24	0.12	0.016	/	/

HC, healthy controls; ARC, allergic rhinoconjunctivitis; AR, allergic rhinitis. / p>0.05.

**Table 5:** The main components of nasal microbiome in the three groups at the Genus level and Metastat analysis results.

Genus	ARC.N Relative abundance(%)	AR.N Relative abundance(%)	H.N Relative abundance(%)	P-value		
				ARC-HC	AR-HC	ARC-AR
Pseudomonas	73.64	70.58	0.71	<0.001	<0.001	/
Ralstonia	3.02	6.54	39.24	<0.001	0.002	/
Serratia	0.02	0.05	4.20	0.006	0.026	/
Chloroplast	0.16	1.24	7.20	0.004	/	/
Corynebacterium	1.60	1.26	9.14	/	0.013	/
Cupriavidus	2.60	1.90	4.56	/	/	/
Mitochondria	0.11	0.23	2.07	/	/	/
Cutibacterium	0.43	0.99	2.48	/	/	/
Muribaculaceae	0.16	0.06	1.73	/	/	/
Moraxella	0.68	1.79	0.00	/	/	/

HC, healthy controls; ARC, allergic rhinoconjunctivitis; AR, allergic rhinitis. / p>0.05.

### Bacterial biomarkers in the three groups

We used the LEfSe algorithm to analyze the structure of the bacterial community associated with the three groups. LEfSe is a high-dimensional algorithm that uses LDA to estimate the effect of differential expression of each taxonomic unit in two groups.

**Figure 6:**
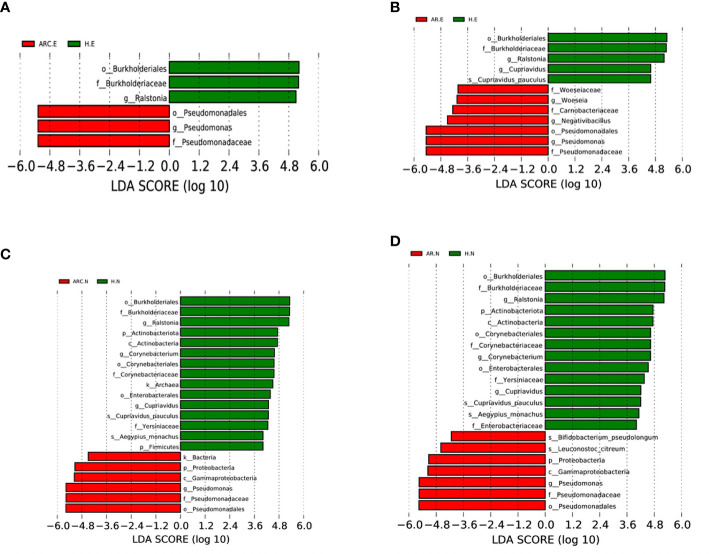
Bacterial biomarkers identified with the linear discriminant analysis effect size (LEfSe) algorithm. Linear discriminant analysis (LDL) scores with the LEfSe tool for taxa, with LDA score > 4 and *P* < 0.05 shown in the histogram. **(A)** Conjunctival bacterial biomarkers identified between ARC group and healthy controls. **(B)** Conjunctival bacterial biomarkers identified between AR group and healthy controls. **(C)** Nasal bacterial biomarkers identified between ARC group and healthy controls. **(D)** Nasal bacterial biomarkers identified between AR group and healthy controls.

With regard to the conjunctival microbiome, at the phylum level, the taxonomic distribution of the three groups was not significantly different. At the genus level, the biomarkers identified were *Pseudomonas* for the ARC group and *Ralstonia* for HCs (**Figure 6A**). Between the AR group and HCs, the genera of the conjunctival microbiome in the AR group were enriched with *Pseudomonas*, *Woeseia*, and *Negativibacillus*. The identified biomarkers for HCs were *Ralstonia* and *Cupriavidus* (**Figure 6B**).

With respect to the nasal microbiome, the phylum biomarker was *Proteobacteria* for the ARC group and AR group, and *Actinobacteriota* and *Firmicutes* for HCs. The most abundant genus in the ARC group and AR group was *Pseudomonas*, whereas *Ralstonia*, *Corynebacterium*, and *Cupriavidus* were enriched in HCs (**Figures 6C, D**).

### Alteration of microbial function

PICRUSt2 was employed to investigate functional alterations in the microbiome of the ARC group and AR group. Compared with that in HCs, functional alteration in the conjunctival microbiome and nasal microbiome in the ARC group and AR group was enormous. With respect to the conjunctival microbiome, among the 10 major metabolic pathways, “gondoate biosynthesis (anaerobic)”, “CDP-diacylglycerol biosynthesis II”, and “CDP-diacylglycerol biosynthesis I” were enriched significantly in the ARC group and AR group. “Aerobic respiration I (cytochrome c)”, and “pyruvate fermentation to isobutanol (engineered)” were predicted to be enriched in HCs. With regard to the nasal microbiome, among the 10 major metabolic pathways, “fatty acid salvage”, “gondoate biosynthesis (anaerobic)”, “CDP-diacylglycerol biosynthesis II”, and “CDP-diacylglycerol biosynthesis I” were enriched significantly in the ARC group and AR group. “Aerobic respiration I (cytochrome c)” and “L-tryptophan biosynthesis” were predicted to be enriched in HCs.

## Discussion

ARC is one of the most common inflammatory disorders. Patients with AR have nasal-allergy symptoms without eye discomfort. Nevertheless, little is known about the microecological differences in the ocular surface and nasal cavity in patients with ARC or AR.

The composition of the microbiota population and host–microbe interactions have key roles in inflammation. Hence, we investigated, for the first time, the microbiota in the ocular surface and nasal cavity in patients with ARC or AR.

Alpha diversity indicates the average species diversity in a given sample. In the present study, the abundance of the conjunctival microbiome in the ARC group and AR group was lower than that in HCs. The diversity of the conjunctival microbiome was significantly lower in the AR group than that in the other two groups. The diversity of the nasal microbiome in the ARC group and AR group was significantly lower than that in HCs. Reduced diversity is considered to be an indicator of unhealthy microbes, and has been associated with different chronic diseases. For instance, reduced diversity of the gut microbiota has been documented in obesity and type-2 diabetes mellitus (33). Hence, reduced diversity indicated an unhealthy conjunctival microbiome and nasal microbiome in patients suffering from ARC or AR. A study on the ocular microbiome of patients with ARC by Yau and colleagues showed no significant change in alpha diversity but, in that study, the Shannon Index in the ocular sample decreased with disease severity (34). Song and coworkers investigated the microbiome of the conjunctival sac in patients with allergic conjunctivitis. They showed that alpha diversity (represented by Observed_species, PD_whole_tree indices, and Chao1 Index) was not significantly different between the normal control group and AC group, but the Shannon Index (which provides information on abundance and homogeneity) was higher in the AC group (35).

Beta diversity denotes the ratio between regional and local species diversity. The “anosim” package revealed significant differences in the community structure of the conjunctival microbiome and nasal microbiome between HCs and patients with ARC or AR, with between-group differences being greater than within-group differences. However, there was no significant difference between the ARC group and AR group in terms of beta diversity.

We showed that the top-four rankings of the three groups for the conjunctival microbiome at the phylum level (as well as the order) were identical, with *Proteobacteria* being the most abundant, followed by *Firmicutes*, *Bacteroidota*, and *Actinobacteriota*. There was no significant difference in abundance between the ARC group and HCs when comparing the abundance of the four main phyla, but the AR group had significantly more *Proteobacteria* and significantly fewer *Actinobacteriota* compared with the HC group. The nasal microbiome of the three groups was enriched with *Proteobacteria* and *Actinobacteriota*, whereas HCs had significantly fewer *Proteobacteria* and significantly more *Actinobacteriota* than patients with ARC or AR. *Proteobacteria* was the most abundant phylum among the three groups in the conjunctiva or nasal cavity, but was less abundant in HCs than in the other two groups.

With respect to the conjunctival microbiome, the genera accounting for >1% in HCs were *Ralstonia*, *Cupriavidus*, *Pseudomonas*, *Bacteroides*, *Faecalibacterium*, *Methyloversatilis*, and *Blautia*. Genera accounting for >1% in the ARC group were *Pseudomonas*, *Ralstonia*, *Cupriavidus*, *Muribaculaceae*, *Methyloversatilis*, *Lactobacillus*, and *Vibrio*. Genera accounting for >1% in the AR group were *Pseudomonas*, *Ralstonia*, and *Cupriavidus*. Although the top-three genera accounted for an identical number of species, there were differences in relative abundance, with significantly more *Pseudomonas* and significantly fewer *Ralstonia* in the ARC group and AR group than in HCs. Among the top-10 genera in the three groups, *Faecalibacterium* and *Prevotella* were significantly less abundant and *Lactobacillus* was significantly more abundant in the ARC group than in HCs. The abundance of *Muribaculaceae* and *Corynebacterium* was significantly lower in the AR group than in HCs. With regard to the nasal microbiome, there was no significant difference between the ARC group and AR group, with *Pseudomonas*, *Ralstonia*, and *Cupriavidus* being the top-three genera. There was a significant difference between HCs and patients with ARC or AR. The abundance of *Pseudomonas* was significantly higher in the ARC group and AR group than in HCs. The abundance of *Ralstonia* and *Serratia* was significantly higher in HCs than in patients with ARC or AR. In the conjunctival microbiome and nasal microbiome, *Pseudomonas* was the most abundant genus in the ARC group and AR group, whereas *Ralstonia* was the most abundant genus in HCs.

We found that the three dominant phyla in the conjunctiva of HCs were *Actinobacteriota*, *Proteobacteria*, and *Firmicutes*. The six genera with the highest relative abundance have been reported to be *Corynebacterium*, *Streptococcus*, *Propionibacterium*, *Bacillus*, *Staphylococcus*, and *Ralsentia* (36). A study of conjunctival bacteria in healthy people found that, whereas there was more obvious transience at OTU and genus levels, greater commonality was observed at the phylum level. Most (94.9%) OTUs were found on the ocular surface in association with three phyla (*Proteobacteria* (64.4%), *Firmicutes* (15.5%), and *Actinobacteriota* (15.0%)), which have also been found to have the highest relative abundance in conjunctiva (18). Song and collaborators found that the five most abundant phyla in the AC group and normal control group were *Firmicutes*, *Proteobacteria*, *Actinobacteriota*, *Bacteroidota*, and *Cyanobacteria*. The five most abundant genera were *Bacillus*, *Staphylococcus*, *Corynebacterium*, *Acinetobacter*, and *Ralstonia* in the AC group and *Acinetobacter*, *Staphylococcus*, *Bacillus*, *Clostridium_sensu_stricto_1*, *Corynebacterium*, and *Geobacillus* in the normal control group (35). Retuerto and coworkers found that 75% of corneal contact lenses adhered to microbiomes in the conjunctiva, skin, and ocular surface after ~30 days of asymptomatic daily wear. *Proteobacteria* was the most abundant phylum, followed by *Firmicutes* and *Actinobacteria*, whereas the most abundant bacterial genera (>1% abundance) were *Ralstonia*, *Enterococcus*, *Streptococcus*, *Halomonas*, *Corynebacterium*, *Staphylococcus*, *Acinetobacter*, *Shewanella*, *Rhodococcus*, and *Cobetia* (37).

Millions of microorganisms reside in the nasal mucosa. It has been reported that microbiome dysbiosis is related to chronic inflammation of nasal mucosa, as observed in AR (38–40) and chronic rhinosinusitis (41), though a clear consensus is lacking. Nevertheless, the effects of the nasal microbiota in the complicated host environment are incompletely understood. Studies have found that the main bacterial phyla in the nasal cavity of healthy humans are *Actinobacteria*, *Firmicutes*, and *Proteobacteria* (42, 43). Gan and colleagues found that the dominant bacterial genus in patients with AR was *Pseudomonas* (44). Yuan and colleagues collected swabs from the inferior turbinate of the nose from patients with AR and HCs. Using high-throughput sequencing of 16S rRNA, they reported no significant difference in the abundance, diversity, or homogeneity of bacterial populations between the AR group and HCs, but the microbiota structure had changed. They observed that the microbiota in the inferior turbinate of patients with AR in the acute-exacerbation stage consisted mainly of the phyla *Proteobacteria*, *Firmicutes*, and *Bacteroidetes*, which was similar to the situation in HCs, but differed in that the abundance of the phylum *Actinobacteria* was increased markedly in patients (45).

Our results and those of other scholars have revealed no significant difference in bacterial species on the ocular surface between patients suffering from allergies and healthy people at the phylum level, but we found differences at the genus level. There are three possible reasons for this observation. First, although all studies used 16S rRNA sequencing, the amplification regions differed among studies, with some studies extending the V1–V3 region and V4 region, whereas we amplified the V3–V4 region. Second, the geographic regions were different; for example, Zhou and colleagues took samples from patients in the Gambia, where sanitary conditions are poor (15), and Song and coworkers took samples from patients in Beijing (35). Third, the age of participants differed; for example, the study by Yau and colleagues was conducted in children with ARC (34).

We found no significant difference in the community structure of conjunctival microbes and nasal microbes between the ARC group and AR group. These data indicated that the structure of the microbial community on the ocular surface was altered by nasal allergy regardless of the presence of ocular allergy. It has been hypothesized that changes in nasal microbes may cause corresponding changes in microbes on the ocular surface. The nasolacrimal duct connects the lacrimal sac to the nasal cavity and plays a part in innate immunity. Thus, “blowing the nose” may encourage nasal bacteria to reach the conjunctiva *via* the nasolacrimal duct in patients suffering from AR. We found that the nasopharyngeal microbiome of ARC patients was similar to the ocular microbiome, whereas the nasopharyngeal microbiome of HCs was significantly different from the ocular microbiome, which suggests a potential interaction between the ocular microbiome and nasal microbiome in these patients (34).

Compared with the microbiome in different parts on the same individual, the microbiome from the same position in the body is similar among different individuals (46). Regardless of abundance, ARC patients appeared to have the same microbiota as that of HCs. Nevertheless, the existence of a “normal” flora population may not denote a healthy state. Bacterial vaginosis is a common example, with imbalance in normal flora inducing changes in pH and overgrowth of specific components of normal flora (47). A similar dysbiosis of ocular flora and nasal flora may be associated with allergies, but further research is needed.

The pathogenesis of allergic diseases is complex and incompletely elucidated. In general, it is believed that allergies occur as a result of chronic inflammation caused by a combination of epithelial cells, intrinsic immunity, and adaptive immunity. In health, T-helper type 1 (Th1) cells/Th2 cells are in balance. However, in patients with allergic diseases, this balance is disturbed, leading to differentiation of T cells towards Th2 cells. Several studies have linked biological disorders to the development of allergic diseases in different anatomic regions (48). The composition and/or imbalance of the microbiome in other sites outside the gut (e.g. lungs, nasopharynx, and nasal cavity) could also have a relationship with allergic diseases. Some studies have linked atopic dermatitis and psoriasis to the abundance of certain bacterial and fungal species, including *Malassezia* species and *Kocuria* species (49–51). However, such studies are in their infancy, few conclusions have been drawn (52), and the role of microbes and how they affect allergic diseases is incompletely understood. Some studies have suggested a link between allergic diseases and the microbiome, but distinguishing between a protective microbiome and one that increases the risk of allergic diseases is difficult (40). However, it is not clear from clinical studies whether changes in the microbiome are the cause or the result of allergic diseases.

The study has some limitations. Firstly, the Shannon curve and the species rarefaction curve showed that the samples collected are representative of the population as a whole. Further study of larger sample sizes or samples collected from different geographic groups or seasons is needed to confirm the consistency of these results. Secondly, clinical parameters included only gender, age and course of disease, and no difference was shown in each subgroup. Subtypes and severity of allergic rhinoconjunctivitis and their relationship to microbiome composition have not been collected and further analyzed. Thirdly, it is important to validate the results by different methods, for example, PCR validation of the main findings of phyla, genus, etc. Future investigations are necessary to validate the conclusions from this study.

## Conclusions

This was a preliminary study to discover if there was ecological dysregulation in patients with ARC or AR compared with HCs. By analyzing alpha diversity, beta diversity, microbiome composition, and their relative abundance, we found changes in the microbiota of the conjunctival sac and nasal cavity. Our data provide deeper understanding of the mechanism responsible for ARC and AR.

## Data availability statement

Publicly available datasets were analyzed in this study. This data can be found here: https://www.ncbi.nlm.nih.gov/ accession number: PRJNA899122.

## Ethics statement

The studies involving human participants were reviewed and approved by the ethics committee of TEDA Hospital (approval 2022-02). The patients/participants provided their written informed consent to participate in this study.

## Author contributions

YW designed the study, collected data, and wrote manuscripts. XL designed the study and revised the manuscript. SG performed the statistical analysis and interpretation of the results. JF collected data. All authors read and approved the final manuscript.

## References

[1] Johansson SG Bieber T Dahl R Friedmann PS Lanier BQ Lockey RF. Revised nomenclature for allergy for global use: Report of the nomenclature review committee of the world allergy organization, October 2003. J Allergy Clin Immunol (2004) 113(5):832–6. doi: 10.1016/j.jaci.2003.12.591 15131563

[2] Bartra J Mullol J Montoro J Jáuregui I del Cuvillos A Dávila I. Effect of bilastine upon the ocular symptoms of allergic rhinoconjunctivitis. J Investig Allergol Clin Immunol (2011) 21 Suppl 3:24–33. 22185047

[3] Shokouhi Shoormasti R Pourpak Z Fazlollahi MR Kazemnejad A Nadali F Ebadi Z. The prevalence of allergic rhinitis, allergic conjunctivitis, atopic dermatitis and asthma among adults of Tehran. Iran J Public Health (2018) 47(11):1749–55. PMC6294865 30581793

[4] Broide DH. The pathophysiology of allergic rhinoconjunctivitis. Allergy Asthma Proc (2007) 28(4):398–403. doi: 10.2500/aap.2007.28.3011 17883906

[5] Ono SJ Abelson MB. Allergic conjunctivitis: update on pathophysiology and prospects for future treatment. J Allergy Clin Immunol (2005) 115(1):118–22. doi: 10.1016/j.jaci.2004.10.042 15637556

[6] Gelardi M Leo ME Quaranta VN Iannuzzi L Tripodi S Quaranta N. Clinical characteristics associated with conjunctival inflammation in allergic rhinoconjunctivitis. J Allergy Clin Immunol Pract (2015) 3(3):387–91. doi: 10.1016/j.jaip.2015.01.006 25634218

[7] Zegans ME Van Gelder RN. Considerations in understanding the ocular surface microbiome. Am J Ophthalmol (2014) 158(3):420–2. doi: 10.1016/j.ajo.2014.06.014 PMC4497523 25132249

[8] Kugadas A Gadjeva M. Impact of microbiome on ocular health. Ocul Surf (2016) 14(3):342–9. doi: 10.1016/j.jtos.2016.04.004 PMC5082109 27189865

[9] Liang Q Li J Zhang S Liao Y Guo S Liang J. Characterization of conjunctival microbiome dysbiosis associated with allergic conjunctivitis. Allergy (2021) 76(2):596–600. doi: 10.1111/all.14635 33080059

[10] Man WH de Steenhuijsen Piters WA Bogaert D. The microbiota of the respiratory tract: gatekeeper to respiratory health. Nat Rev Microbiol (2017) 15(5):259–70. doi: 10.1038/nrmicro.2017.14 PMC7097736 28316330

[11] Strachan DP. Hay fever, hygiene, and household size. BMJ (1989) 299(6710):1259–60. doi: 10.1136/bmj.299.6710.1259 PMC1838109 2513902

[12] Willcox MD. Characterization of the normal microbiota of the ocular surface. Exp Eye Res (2013) 117:99–105. doi: 10.1016/j.cmi.2019.05.011 23797046

[13] Shovlin JP Argüeso P Carnt N Chalmers RL Efron N Fleiszig SM. Ocular surface health with contact lens wear. Cont Lens Anterior Eye (2013) 36 Suppl 1:S14–21. doi: 10.1016/S1367-0484(13)60005-3 23347571

[14] Doan T Akileswaran L Andersen D Johnson B Ko N Shrestha A. Paucibacterial microbiome and resident DNA virome of the healthy conjunctiva. Invest Ophthalmol Vis Sci (2016) 57(13):5116–26. doi: 10.1167/iovs.16-19803 PMC5054734 27699405

[15] Zhou Y Gao H Mihindukulasuriya KA La Rosa PS Wylie KM Vishnivetskaya T. Biogeography of the ecosystems of the healthy human body. Genome Biol (2013) 14(1):R1. doi: 10.1186/gb-2013-14-1-r1 23316946 PMC4054670

[16] Lee SH Oh DH Jung JY Kim JC Jeon CO. Comparative ocular microbial communities in humans with and without blepharitis. Invest Ophthalmol Vis Sci (2012) 53(9):5585–93. doi: 10.1167/iovs.12-9922 22836761

[17] Graham JE Moore JE Jiru X Moore JE Goodall EA Dooley JS. Ocular pathogen or commensal: a PCR-based study of surface bacterial flora in normal and dry eyes. Invest Ophthalmol Vis Sci (2007) 48(12):5616–23. doi: 10.1167/iovs.07-0588 18055811

[18] Dong Q Brulc JM Iovieno A Bates B Garoutte A Miller D. Diversity of bacteria at healthy human conjunctiva. Invest Ophthalmol Vis Sci (2011) 52(8):5408–13. doi: 10.1167/iovs.10-6939 PMC3176057 21571682

[19] Shin H Price K Albert L Dodick J Park L Dominguez-Bello MG. Changes in the eye microbiota associated with contact lens wearing. mBio (2016) 7(2):e00198. doi: 10.1128/mBio.00198-16 27006462 PMC4817251

[20] Ozkan J Willcox M Wemheuer B Wilcsek G Coroneo M Thomas T. Biogeography of the human ocular microbiota. Ocul Surf (2019) 17(1):111–8. doi: 10.1016/j.jtos.2018.11.005 30445178

[21] Han X Krempski JW Nadeau K. Advances and novel developments in mechanisms of allergic inflammation. Allergy (2020) 75(12):3100–11. doi: 10.1111/all.14632 33068299

[22] Huang YJ Marsland BJ Bunyavanich S O'Mahony L Leung DY Muraro A. The microbiome in allergic disease: Current understanding and future opportunities-2017 PRACTALL document of the American academy of allergy, asthma & immunology and the European academy of allergy and clinical immunology. J Allergy Clin Immunol (2017) 139(4):1099–110. doi: 10.1016/j.jaci.2017.02.007 PMC5899886 28257972

[23] Huang YJ Nariya S Harris JM Lynch SV Choy DF Arron JR. The airway microbiome in patients with severe asthma: Associations with disease features and severity. J Allergy Clin Immunol (2015) 136(4):874–84. doi: 10.1016/j.jaci.2015.05.044 PMC4600429 26220531

[24] Lynch SV. Gut microbiota and allergic disease. New Insights Ann Am Thorac Soc (2016) 13 Suppl 1(Suppl 1):S51–4. doi: 10.1513/AnnalsATS.201507-451MG PMC5015732 27027953

[25] Yang HJ LoSavio PS Engen PA Naqib A Mehta A Kota R. Association of nasal microbiome and asthma control in patients with chronic rhinosinusitis. Clin Exp Allergy (2018) 48(12):1744–7. doi: 10.1111/cea.13255 PMC6265059 30126004

[26] Watters GA Turnbull PR Swift S Petty A Craig JP. Ocular surface microbiome in meibomian gland dysfunction. Clin Exp Ophthalmol (2017) 45(2):105–11. doi: 10.1111/ceo.12810 27473509

[27] Brożek JL Bousquet J Agache I Agarwal A Bachert C Bosnic-Anticevich S. Allergic rhinitis and its impact on asthma (ARIA) guidelines-2016 revision. J Allergy Clin Immunol (2017) 140(4):950–8. doi: 10.3760/cma.j.issn.1673-0860.2018.10.017 28602936

[28] Sánchez-Hernández MC Montero J Rondon C Benitez del Castillo JM Velázquez E Herreras JM. SEAIC 2010 rhinoconjunctivitis committee; Spanish group ocular surface-GESOC. consensus document on allergic conjunctivitis (DECA). J Investig Allergol Clin Immunol (2015) 25(2):94–106. 25997302

[29] Magoč T Salzberg SL. FLASH: fast length adjustment of short reads to improve genome assemblies. Bioinformatics (2011) 27(21):2957–63. doi: 10.1093/bioinformatics/btr507 PMC3198573 21903629

[30] Haas BJ Gevers D Earl AM Feldgarden M Ward DV Giannoukos G. Chimeric 16S rRNA sequence formation and detection in Sanger and 454-pyrosequenced PCR amplicons. Genome Res (2011) 21:494–504. doi: 10.1101/gr.112730.110 21212162 PMC3044863

[31] Caporaso JG Kuczynski J Stombaugh J Bittinger K Bushman FD Costello EK. QIIME allows analysis of high-throughput community sequencing data. Nat Methods (2010) 7(5):335–6. doi: 10.1038/nmeth.f.303 PMC3156573 20383131

[32] Li MJ Shao DT Zhou JC Gu JH Qin JJ Chen W. Signatures within esophageal microbiota with progression of esophageal squamous cell carcinoma. Chin J Cancer Res (2020) 32:755–67. doi: 10.21147/j.issn.1000-9604.2020.06.09 PMC7797230 33446998

[33] Deng F Li Y Zhao J. The gut microbiome of healthy long-living people. Aging (Albany NY) (2019) 11(2):289–90. doi: 10.18632/aging.101771 PMC6366966 30648974

[34] Yau JW Hou J Tsui SKW Leung TF Cheng NS Yam JC. Characterization of ocular and nasopharyngeal microbiome in allergic rhinoconjunctivitis. Pediatr Allergy Immunol (2019) 30(6):624–31. doi: 10.1111/pai.13088 31132163

[35] Song H Xiao K Min H Chen Z Long Q. Characterization of conjunctival sac microbiome from patients with allergic conjunctivitis. J Clin Med (2022) 11(4):1130. doi: 10.3390/jcm11041130 35207407 PMC8875969

[36] Zhou Y Holland MJ Makalo P Joof H Roberts CH Mabey DC. The conjunctival microbiome in health and trachomatous disease: a case control study. Genome Med (2014) 6(11):99. doi: 10.1186/s13073-014-0099-x 25484919 PMC4256740

[37] Retuerto MA Szczotka-Flynn L Mukherjee PK Debanne S Iyengar SK Richardson B. Diversity of ocular surface bacterial microbiome adherent to worn contact lenses and bacterial communities associated with care solution use. Eye Contact Lens (2019) 45(5):331–9. doi: 10.1097/ICL.0000000000000578 30724840

[38] Chiu CY Chan YL Tsai YS Chen SA Wang CJ Chen KF. Airway microbial diversity is inversely associated with mite-sensitized rhinitis and asthma in early childhood. Sci Rep (2017) 7(1):1820. doi: 10.1038/s41598-017-02067-7 28500319 PMC5431806

[39] Hyun DW Min HJ Kim MS Whon TW Shin NR Kim PS. Dysbiosis of inferior turbinate microbiota is associated with high total IgE levels in patients with allergic rhinitis. Infect Immun (2018) 86(4):e00934–e1017. doi: 10.1128/IAI.00934-17 PMC5865026 29426044

[40] Ipci K Altıntoprak N Muluk NB Senturk M Cingi C. The possible mechanisms of the human microbiome in allergic diseases. Eur Arch Otorhinolaryngol (2017) 274(2):617–26. doi: 10.1007/s00405-016-4058-6 27115907

[41] Gan W Yang F Tang Y Zhou D Qing D Hu J. The difference in nasal bacterial microbiome diversity between chronic rhinosinusitis patients with polyps and a control population. Int Forum Allergy Rhinol (2019) 9(6):582–92. doi: 10.1007/s00405-020-06311-1 30720930

[42] Yan M Pamp SJ Fukuyama J Hwang PH Cho DY Holmes S. Nasal microenvironments and interspecific interactions influence nasal microbiota complexity and s. aureus carriage. Cell Host Microbe (2013) 14(6):631–40. doi: 10.1016/j.chom.2013.11.005 PMC3902146 24331461

[43] Frank DN Feazel LM Bessesen MT Price CS Janoff EN Pace NR. The human nasal microbiota and staphylococcus aureus carriage. PloS One (2010) 5(5):e10598. doi: 10.1371/journal.pone.0010598 20498722 PMC2871794

[44] Gan W Yang F Meng J Liu F Liu S Xian J. Comparing the nasal bacterial microbiome diversity of allergic rhinitis, chronic rhinosinusitis and control subjects. Eur Arch Otorhinolaryngol (2021) 278(3):711–8. doi: 10.1007/s00405-020-06311-1 32860131

[45] Yuan Y Wang C Wang G Guo X Jiang S Zuo X. Airway microbiome and serum metabolomics analysis identify differential candidate biomarkers in allergic rhinitis. Front Immunol (2022) 12:771136. doi: 10.3389/fimmu.2021.771136 35069544 PMC8766840

[46] Sonnenburg JL Fischbach MA. Community health care: therapeutic opportunities in the human microbiome. Sci Transl Med (2011) 3(78):78ps12. doi: 10.1126/scitranslmed.3001626 PMC3287364 21490274

[47] Turovskiy Y Sutyak Noll K Chikindas ML. The aetiology of bacterial vaginosis. J Appl Microbiol (2011) 110(5):1105–28. doi: 10.1111/j.1365-2672.2011.04977.x PMC3072448 21332897

[48] Cavuoto KM Stradiotto AC Galor A. Role of the ocular surface microbiome in allergic disease. Curr Opin Allergy Clin Immunol (2019) 19(5):482–7. doi: 10.1097/ACI.0000000000000559 PMC13461304 31169596

[49] Stehlikova Z Kostovcik M Kostovcikova K Kverka M Juzlova K Rob F. Dysbiosis of skin microbiota in psoriatic patients: Co-occurrence of fungal and bacterial communities. Front Microbiol (2019) 10:438. doi: 10.3389/fmicb.2019.00438 30949136 PMC6437110

[50] Sei Y. [Malassezia related diseases]. Med Mycol J (2012) 53(2):97–102. doi: 10.3314/mmj.53.97 22728591

[51] Bjerre RD Bandier J Skov L Engstrand L Johansen JD. The role of the skin microbiome in atopic dermatitis: a systematic review. Br J Dermatol (2017) 177(5):1272–8. doi: 10.1111/bjd.15390 28207943

[52] Lan F Zhang N Gevaert E Zhang L Bachert C. Viruses and bacteria in Th2-biased allergic airway disease. Allergy (2016) 71:1381–92. doi: 10.1111/all.12934 27188632

